# TIPE regulates VEGFR2 expression and promotes angiogenesis in colorectal cancer

**DOI:** 10.7150/ijbs.37906

**Published:** 2020-01-01

**Authors:** Mengya Zhong, Nini Li, Xingfeng Qiu, Yuhan Ye, Huiyu Chen, Jianyu Hua, Ping Yin, Guohong Zhuang

**Affiliations:** 1 Cancer Research Center, School of Medicine, Xiamen University, Xiamen, Fujian, China.; 2 Department of Pathology, The First Affiliated Hospital of Yangtze University, Jingzhou, Hubei, China.; 3 Department of Gastrointestinal Surgery, Zhongshan Hospital Affiliated to Xiamen University, Xiamen, Fujian, China.; 4 Department of Pathology, Zhongshan Hospital Affiliated to Xiamen University, Xiamen, Fujian, China.; 5 Organ Transplantation Institute of Xiamen University, Fujian Provincial Key Laboratory of Organ and Tissue Regeneration, School of Medicine, Xiamen University, Xiamen, Fujian, China.

**Keywords:** CRC, TIPE, angiogenesis, VEGFR2, PDK1, PI3K-Akt

## Abstract

**Background:** Metastasis is the leading cause of death in colorectal cancer (CRC) patients. It is regulated mainly by tumor cell angiogenesis, and angiogenesis is caused by the binding of vascular endothelial growth factor (VEGF) to vascular endothelial growth factor receptor 2 (VEGFR2). Tumor necrosis factor-α-induced protein 8 (TNFAIP8, hereto after TIPE) plays an important role in tumorigenesis, development, and prognosis. However, the relationship between TIPE and VEGFR2 in CRC angiogenesis and the mechanism of action remain unknown.

**Method:** In this study, we used quantitative real-time PCR, Western blotting and immunohistochemistry to detect TIPE and VEGFR2 expression in 55 specimens from CRC patients. We also used HCT116 CRC cells and human umbilical vein endothelial cells (HUVECs) for in vitro experiments by stably transducing shTIPE and shRNA control lentivirus into HCT116 cells, detecting VEGFR2 expression after TIPE knockdown and repurposing the culture supernatant as conditioned medium to stimulate angiogenesis of HUVECs. In vivo experiments with chicken chorioallantoic membranes (CAMs) and a nude mouse matrix subcutaneous tumor model were performed to validate the effects of TIPE on angiogenesis. Additionally, we analyzed the expression and phosphorylation levels of PDK1 and blocked PDK1 expression using inhibitors to determine whether TIPE-induced changes in VEGFR2-mediated angiogenesis acted via the PI3K-Akt pathway.

**Results:** We found that TIPE and VEGFR2 are highly expressed in CRC and act as oncogenes. TIPE knockdown also downregulated VEGFR2 expression, which resulted in simultaneous inhibition of cell proliferation, cell migration and angiogenesis. Then, in vivo experiments further demonstrated that TIPE promotes angiogenesis in CRC. Finally, we found that TIPE promotes VEGFR2-mediated angiogenesis by upregulating PDK1 expression and phosphorylation and that blocking PDK1 expression can inhibit this process.

**Conclusion:** TIPE promotes angiogenesis in CRC by regulating the expression of VEGFR2, which may be a target for antiangiogenic cancer therapy.

## Introduction

Colorectal cancer (CRC) is a malignant tumor that seriously endangers human health and is the fourth most common cancer, just below breast cancer, prostate cancer, and lung cancer. According to GLOBOCAN 2018 data (http://gco.iarc.fr.), there were approximately 500,000 new cases of CRC in China, accounting for 28% of new cases worldwide, and nearly 300,000 deaths, accounting for 34% of global deaths; these data suggest that China has the highest CRC morbidity and mortality rates in the world 1. Although surgery, radiotherapy, chemotherapy, targeted therapy, and immunotherapy have curative effects, the long-term survival of patients with CRC is still low due to high rates of recurrence and metastasis; the occurrence of postoperative metastasis, which is the main cause of death in patients with CRC, is particularly high 2, 3. Moreover, radiotherapy and chemotherapy elicit serious adverse reactions, and secondary chemotherapy resistance is very common 4. Therefore, novel therapeutic strategies for the treatment of CRC and a better understanding of the molecular mechanisms of CRC metastasis are urgently needed.

The tumor necrosis factor-α-induced protein 8 (TNFAIP8) families is a recently identified protein family that has been reported to play a crucial role in immune homeostasis, inflammatory responses, tumorigenesis, progression, and signal transduction 5, 6. The family consists of four members: TNFAIP8 (TIPE) 7, TNFAIP8L1 (TIPE1) 8, TNFAIP8L2 (TIPE2) 9, and TNFAIP8L3 (TIPE3) 10. TIPE, also known as SCC-S2, GG2-1, NDED, and MDC-3.13, is a 23-kDa cytoplasmic protein with a small death-effector domain (DED) at the amino terminus that is homologous to the DED II domain on FLIP. TIPE was also the first discovered member and was originally identified as a partial cDNA clone in head and neck squamous cell carcinoma (HNSCC) cells in the late 1990s; these cells were derived from patients with metastatic radioresistant HNSCC 11, 12. TIPE is currently the most studied protein of the TNFAIP8 family, and related research has shown that it can regulate tumor apoptosis, tumorigenesis, progression, and prognosis 13, 14. In recent years, many studies have found that TIPE is highly expressed in cervical cancer 15, ovarian cancer 16, 17, breast cancer 18, 19, gastric cancer 20, 21, non-small-cell lung cancer 22, pancreatic cancer 23, endometrial cancer 24 and papillary thyroid carcinoma 25. Likewise, it is correlated with corresponding clinicopathological characteristics, treatment, and prognosis. The most recent studies have shown that TIPE is overexpressed and regulates tumor cell proliferation in CRC 26, but the mechanisms related to the role of TIPE in angiogenesis in CRC have not been clarified. Identifying biomolecules directly involved in tumor metastasis and cell survival is an important step in the rational design of therapeutic drugs for advanced malignancies.

Angiogenesis is one of the most important steps in tumor progression. The formation of new blood vessels provides nutrients and oxygen to the tumor, consequently promoting the rapid proliferation of cancer cells. Half of patients with CRC die from local spread, and the other half die from distant metastasis 27. Metastasis is the process by which cancer cells spontaneously spread to secondary sites and heavily relies on angiogenesis 28. Cancer cells release large amounts of angiogenic molecules that induce the expression of angiogenic receptors in tumor blood vessels (e.g., endothelial growth factor (EGF) induces EGF receptors and vascular endothelial growth factor (VEGF) induces VEGF receptors (VEGFRs) in tumor-associated blood vessels) 29. VEGF-A is a key mediator of angiogenesis and is induced by the family of tyrosine kinase receptors containing VEGFRs 30. Although VEGF-A ligand binds to VEGFR1 and VEGFR2, the signal mainly transduces through VEGFR2, leading to vascular permeability and endothelial cell proliferation, survival, and migration 31. These pathophysiological changes involve the initiation of multiple signal transduction pathways, such as the phosphoinositide 3-kinase (PI3K)-Akt, mitogen-activated protein kinase (MAPK), and calcium signaling pathways, to promote the activation of VEGF signaling pathways 32. Early studies have illustrated the effect of TIPE on the regulation of VEGFR2 in breast cancer metastasis 19. Recent studies have found that the presence of VEGFR protein in tissues and serum is associated with CRC metastasis 33. Therefore, although there are data stating that TIPE and VEGFR2 are involved in the metastasis processes of most cancers, their involvement in CRC angiogenesis and the mechanisms involved remain elusive.

In this study, we investigated the role of TIPE and VEGFR2 in tumor angiogenesis in CRC. We found that TIPE and VEGFR2 were highly expressed in 55 human CRC samples. Through in vivo and in vitro experiments, we also found that the loss of TIPE affected VEGFR2 expression and inhibited tumor-based angiogenesis. At the same time, TIPE promoted angiogenesis in CRC by upregulating the expression and phosphorylation of PDK1, suggesting that TIPE and VEGFR2 are both involved in the process of angiogenesis in CRC and are potential targets for CRC treatment.

## Results

### TIPE and VEGFR2 are highly expressed in CRC and act as oncogenes

**Figure 1:**
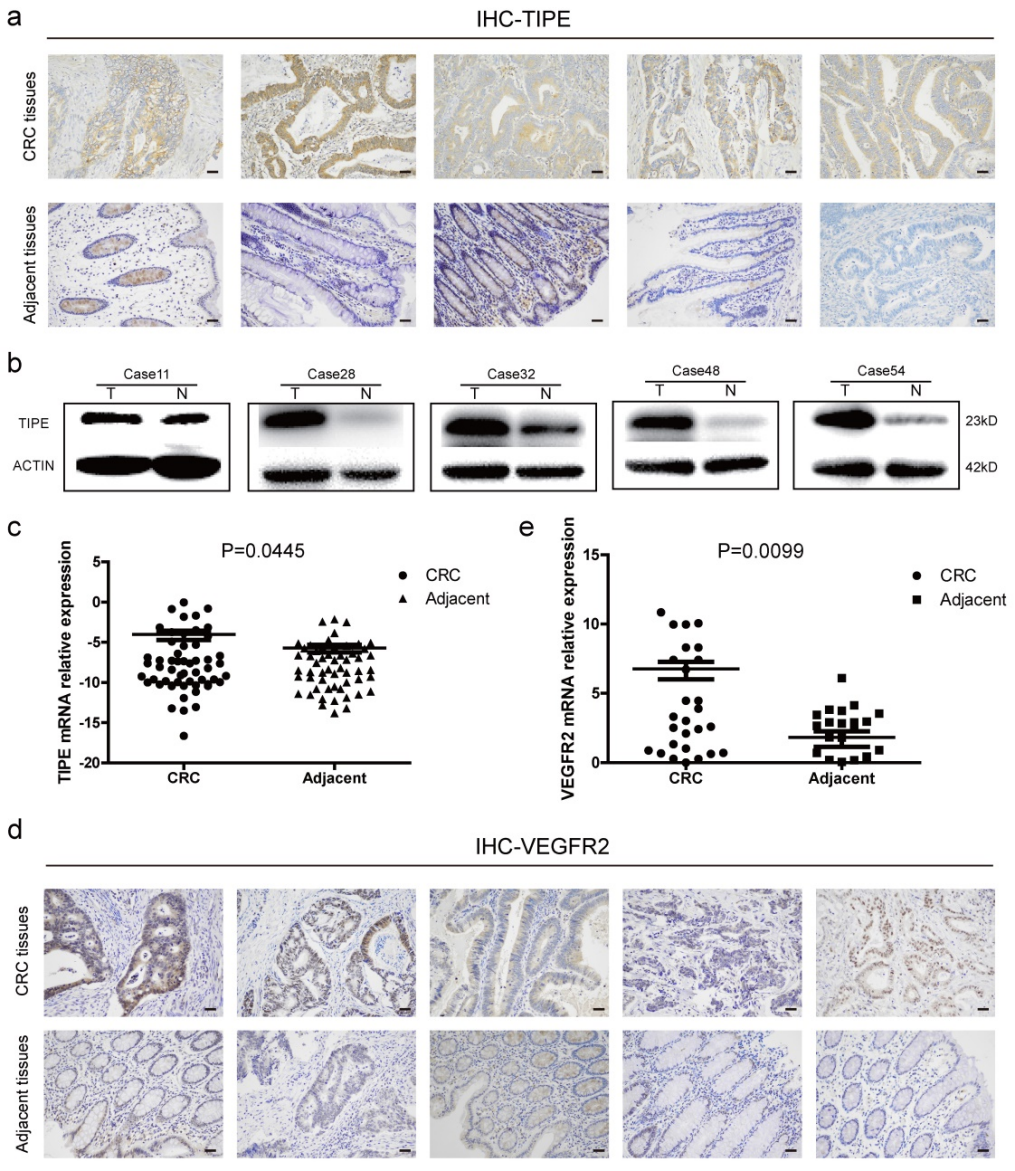
Tumor necrosis factor-α induced protein 8 (TIPE) and vascular endothelial growth factor receptor 2 (VEGFR2) are highly expressed in CRC and act as oncogenes. **(a)** TIPE expression in five pairs of fresh human CRC tumor tissues and matched adjacent tissues analyzed by immunohistochemistry staining. **(b)** Representative results of TIPE expression in five pairs of fresh CRC tumor tissues and adjacent tissues as detected by Western blot; T indicates tumor tissue, and N indicates normal tissue. **(c)** Comparison of TIPE mRNA expression levels in human CRC tissues and matched adjacent tissues. TIPE mRNA expression was quantified by qRT-PCR and normalized to that in the matched adjacent normal tissues. **(d)** Through immunohistochemical staining, VEGFR2 expression was analyzed in five randomly selected pairs of CRC tumor tissues and matched adjacent tissues. **(e)** Comparison of VEGFR2 mRNA expression levels in human CRC tissues and matched adjacent tissues. VEGFR2 mRNA expression was quantified by qRT-PCR and normalized to that in the matched adjacent normal tissues.

To determine the role of TIPE in CRC, we collected fresh tissue biopsies from patients who were clinically diagnosed with CRC. First, we examined TIPE protein expression in five random CRC patients and matched adjacent tissues. According to the immunohistochemistry staining, as shown in Figure 1a and Supplementary Figure 1a, TIPE was higher in CRC tissues than in adjacent tissues, and subsequent Western blotting demonstrated that TIPE expression was strongly upregulated in CRC tissues (Figure 1b). Next, we expanded the cohort size to 55 patients and detected TIPE expression by quantitative real-time PCR. The results indicate that relative TIPE mRNA expression was also significantly higher in tumor tissues (n = 29) than in adjacent tissues (n = 26) (Figure 1c). To determine whether the increased TIPE expression was associated with clinical characteristics, we analyzed the sex, age, invasion depth, lymph node metastasis, distant metastasis and staging of CRC patients and observed that TIPE expression was higher in the muscularis propria and lymph node metastasis group than in the no membrane invasion and lymph node metastasis group (p< 0.05), but there was no significant correlation with sex, age, distant metastasis or staging (p>0.05) (Table 1).

Because VEGFR2 has been indicated as a key factor of angiogenesis in many previous studies, we used Oncomine, a classic database of differentially expressed tumor genes, to query the expression of TIPE and VEGFR2 in CRC and determine whether there is a relationship between them. After conducting the analysis, we found that TIPE and VEGFR2 are highly expressed in CRC (Supplementary Figure 1b, 1c), and a linear analysis showed a positive correlation (Supplementary Figure 1d). We then randomly selected ten clinical samples to detect VEGFR2 expression by immunohistochemistry and found that VEGFR2 expression was higher in CRC tissues than in healthy adjacent tissues (Figure 1d, Supplementary Figure 1f). To verify the correlation between VEGFR2 and TIPE, we examined VEGFR2 expression in the 55 CRC specimens by qRT-PCR, and the results showed that the relative VEGFR2 mRNA expression was also significantly higher in tumor tissues (n = 36) than in adjacent tissues (n = 19) (Figure 1e). We compared the difference between TIPE and VEGFR2 overexpression in CRC, and it proved to be statistically significant (Supplementary Table 1). The results from the clinical sample assessments are consistent with our Oncomine analysis, indicating that the mRNA and protein expression levels of TIPE and VEGFR2 are simultaneously higher in CRC tissues than in adjacent tissues and that the differential expression of TIPE is associated with poor prognosis, suggesting that TIPE and VEGFR2 play roles as oncogenes in CRC.

### Decreasing TIPE expression can downregulate VEGFR2 expression and inhibit cell proliferation, cell migration, and angiogenesis

**Figure 2:**
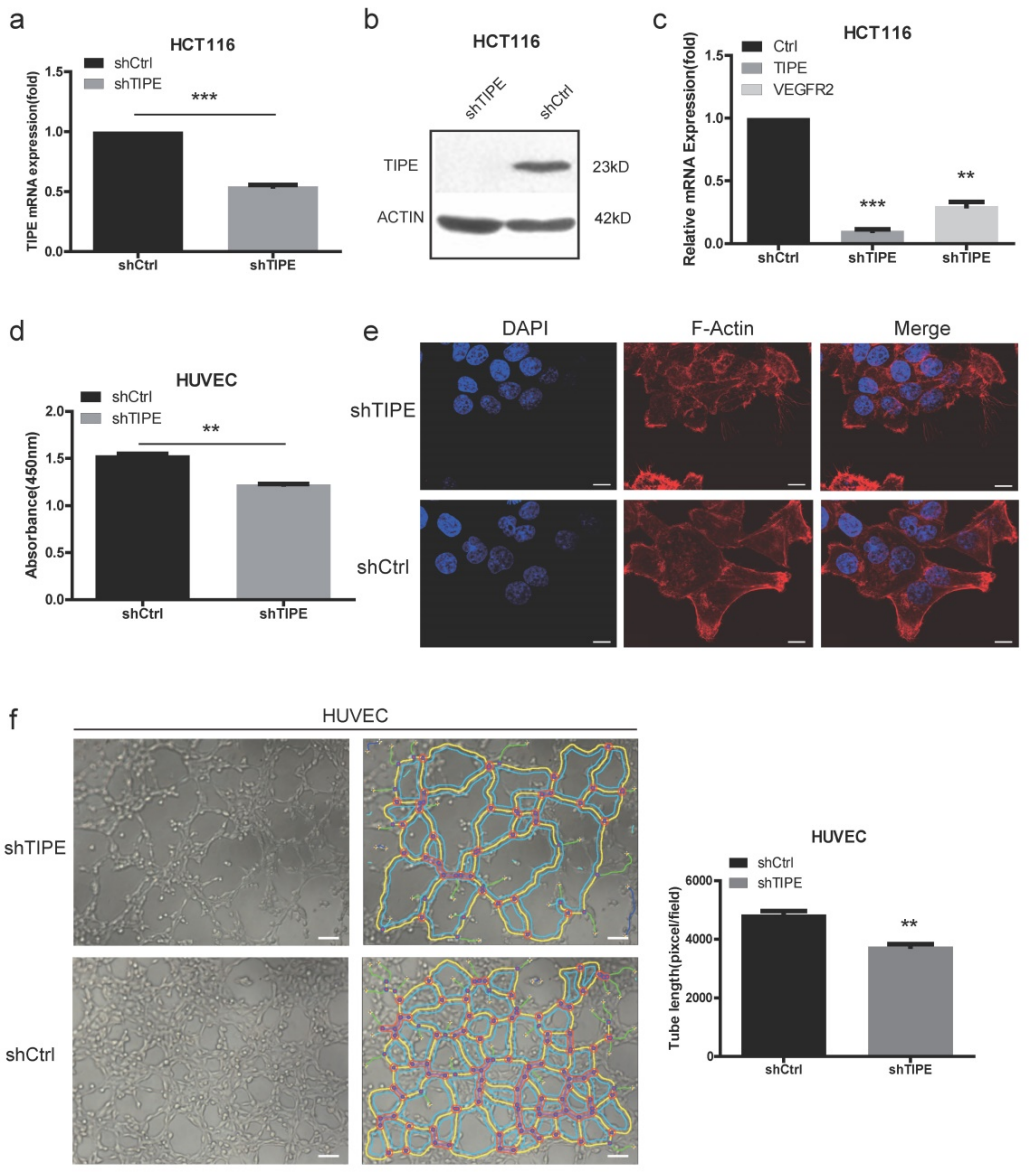
Knockdown of TIPE downregulates VEGFR2 expression and inhibits cell proliferation, cell migration and angiogenesis. **(a)** qRT-PCR of TIPE expression in HCT116 cells infected with lentiviral shTIPE or control shRNA. TIPE mRNA expression was quantified by qRT-PCR and normalized to that in the shRNA control cells. **(b)** TIPE expression in HCT116 cells infected with lentiviral shTIPE or control shRNA according to Western blot analysis. **(c)** Expression of VEGFR2 in shTIPE and shRNA control HCT116 cells based on qRT-PCR assays. **(d)** Tumor cell culture conditioned medium (TCM) from HCT116 cells stably transduced with shTIPE or control shRNA inhibited the proliferation of HUVECs as determined by CCK-8 assays. **(e)** Representative immunofluorescence (IF) images demonstrated that the level of TIPE has an effect on the expression of microfilaments and initial pseudopod extension in the stably transduced HCT116 cell lines. Scale bar, 50 µm. **(f)** Representative images (left panel) and quantification (right panel) of tube formation of HUVECs treated with TCM derived from control HCT116 cells or HCT116 cells with stable TIPE knockdown. The data were collected from four independent experiments using different batches of cells. Scale bar, 500 μm. **p <0.01, ***p<0.001.

To investigate the potential cellular functions of TIPE in CRC, we stably transduced HCT116 cells with shTIPE or control shRNA. qRT-PCR and Western blot analyses were performed to measure TIPE expression in the transduced HCT116 cells, and shTIPE was more efficient at reducing mRNA and protein levels than was the control shRNA (Figure 2a, 2b). To further clarify the regulation of VEGFR expression by TIPE, we used qRT-PCR to detect the expression level of VEGFR2 in shTIPE HCT116 cells and shRNA control HCT116 cells and found that VEGFR2 mRNA expression was lower in the shTIPE group than in the control group, indicating that knockdown of TIPE can inhibit VEGFR2 expression (Figure 2c). Then, we used functional assays to determine whether TIPE affects VEGFR2-mediated angiogenesis.

Because proliferation, migration, and angiogenesis are critical steps in tumor growth and metastasis, we cultured HUVECs, a cell line whose migration is crucial for angiogenesis, in conditioned medium (TCM) collected from shTIPE or shRNA control HCT116 cells. We used CCK-8 assays to test the effect of the media on HUVEC proliferation and discovered that it was significantly inhibited in the shTIPE group compared with that in the control group (Figure 2d). Dynamic cytoskeletal changes can promote the mobility of cancer cells, making it easier for them to migrate to different sites and thus further facilitate angiogenesis 34. Microfilaments are important components of the cytoskeleton, and thick microfilaments promote the migration of cancer cells. We performed immunofluorescence (IF) staining using shTIPE and shRNA control HCT116 cells. After the cells were staining with phalloidin red, it was clear by laser scanning confocal microscopy that the shTIPE group had less microfilament distribution in the cell membrane and weaker red fluorescent signals than the control group, indicating that the control group but not the shTIPE group had extended pseudopods (Figure 2e).

Then, we seeded HUVECs into a 96-well plate coated with Matrigel and cultured them with TCM for 3-6 hours. Next, three to five regional microscope fields were randomly selected. After the total length of the branches was counted, it was found that the branches of the shTIPE experimental group were shorter than those of the control group. This indicates that TCM from shTIPE cells significantly inhibited tube formation ability compared to that of TCM from shRNA control cells (Figure 2f). These results confirm that knocking down TIPE can downregulate VEGFR2 expression, which consequently inhibits cell proliferation, migration, and angiogenesis.

### TIPE promotes CRC angiogenesis in vivo

**Figure 3:**
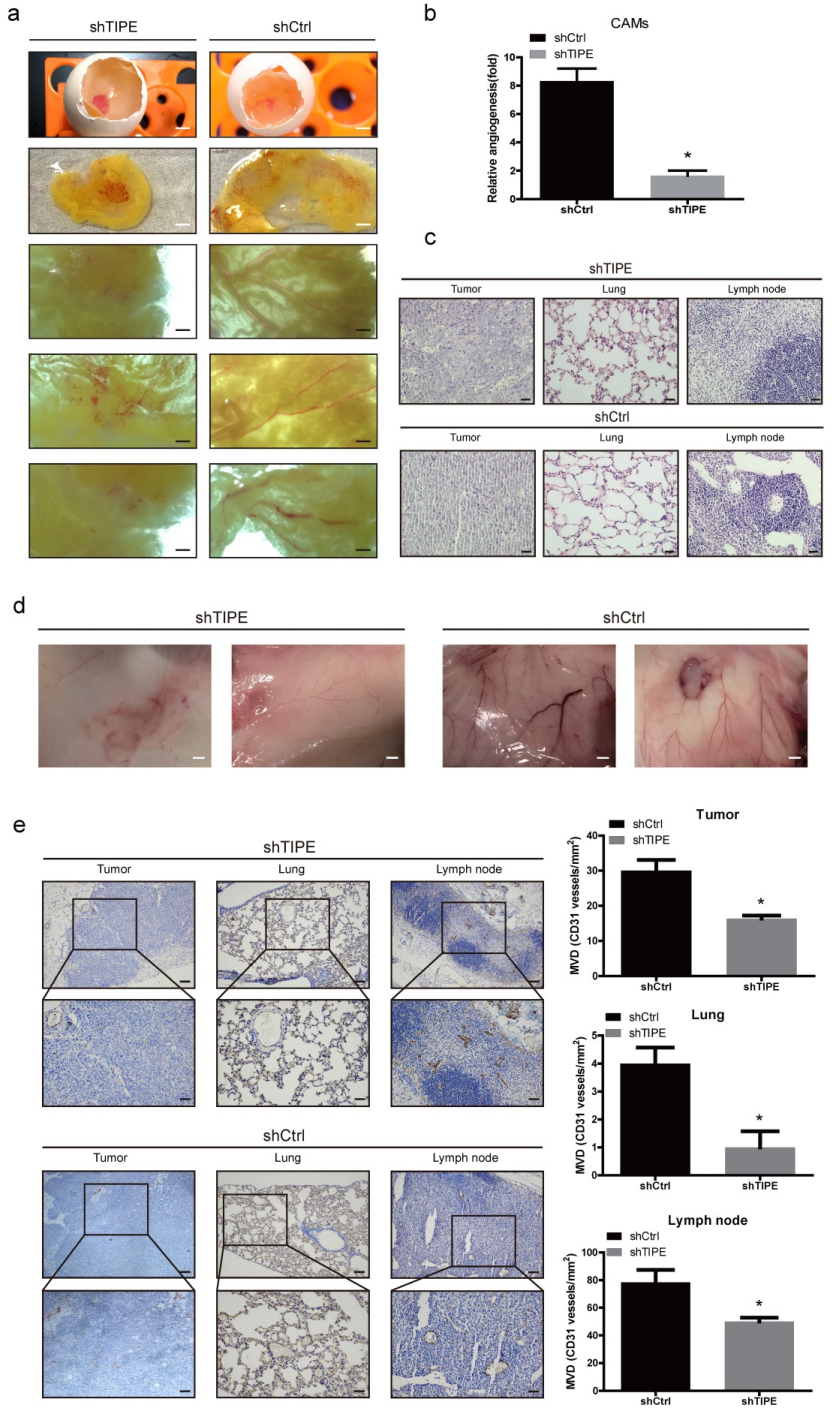
** Effects of TIPE on tumor angiogenesis in vivo. (a)** Cells were mixed with Matrigel and subsequently implanted onto chicken chorioallantoic membranes (CAMs), as described in the Materials and Methods. Representative images of angiogenesis on the CAMs are shown. The physical figure scale bar is 1 mm, and the somatic microscope scale bar is 10 µm. **(b)** The number of blood vessels was normalized to that of the respective control group, and the results are expressed as the means ± SEM (n = 5, right). **(c)** Hematoxylin and eosin (HE) staining analysis of histological features in the plug tissues, lungs, and lymph nodes of nude mice. Scale bar, 100 µm. **(d)** Cells were mixed with Matrigel and injected into the right flanks of nude mice. Seven days after implantation, the gel plugs were collected and photographed. Scale bar, 10 µm. **(e)** Immunohistochemical staining analysis of the levels of CD31 in the plug tissues from nude mice. The right panel shows the quantification of CD31-positive vessels. Scale bar, 200 µm (above), 100 µm (below), * p<0.05.

During the process of pathological angiogenesis, tumor cells enter the systemic circulation from the primary site by invading the blood vessel wall. Vascular density can be used as a reference indicator for tumor metastasis potential. The higher the tumor vascular development level is, the higher the incidence of metastasis 35. Chicken chorioallantoic membrane (CAM) assays have proven to be a unique in vivo model for studying the process of neovascularization and the effects of antiangiogenic drugs. To confirm the consistency of the in vitro results, we evaluated the effect of TIPE on angiogenesis in the CAM model. As shown in Figure 3a, the blood vessels in the control CAM formed successfully and were accompanied by multiple branches, while the shTIPE CAM had only blurred spots and no blood vessel formation. Likewise, TIPE knockdown reduced the angiogenesis index by 0.25-fold compared with the control conditions (Figure 3b).

To further validate these results, we subcutaneously injected a mixture of Matrigel with the constructed HCT116 cell lines into nude mice to form a Matrigel plug, which we used to investigate the role of TIPE in angiogenesis. On the ninth day after injection, we removed the gel plugs and photographed them; then, the gel plugs were subjected to hematoxylin and eosin (HE) and immunohistochemical staining. According to the HE staining results, the shTIPE group showed clear cell structures and deep nuclear staining, and lymphocyte infiltration around the cancer tissue was not obvious. The pulmonary septum was widened with alveolar edema with lymphocyte infiltration. In contrast, the shCtrl group showed lightly stained nuclei, blurred nuclear structure, and unclear boundaries, and the pulmonary septum was hyperemic and widened with extensive lymphocyte infiltration. Both lymph nodes had considerable inflammatory cell infiltration. These findings indicate that the shCtrl group had a more pronounced degree of malignancy (Figure 3c). In this model, it was revealed that TIPE knockdown significantly reduced angiogenesis, while the TIPE control group exhibited significant formation of new blood vessels in the embolus as determined by the microvessel density (Figure 3d). Moreover, the expression level of CD31 (a marker of blood vessels) was increased in the TIPE control group (Figure 3e). These results indicate that TIPE also promotes angiogenesis in vivo.

### TIPE promotes VEGFR2-mediated angiogenesis by upregulating PDK1 expression and phosphorylation

**Figure 4:**
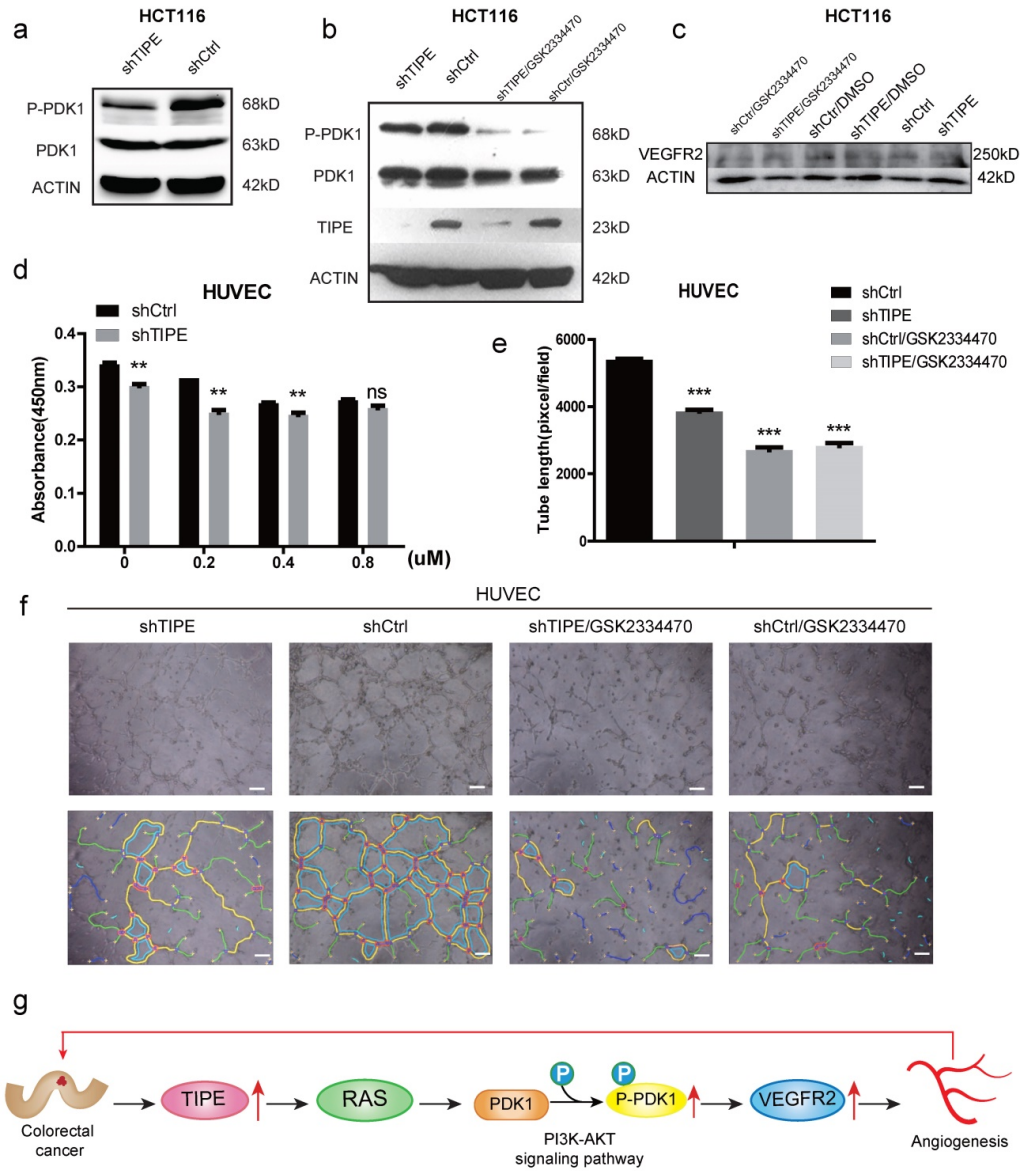
** TIPE promotes VEGFR2-mediated angiogenesis by upregulating PDK1 expression and phosphorylation. (a)** TIPE knockdown in HCT116 cells inhibits PDK1 phosphorylation according to Western blot analysis.** (b)** After cells were treated with a PDK1 inhibitor (GSK2334470), the expression levels of TIPE and PDK1 and their corresponding phosphorylation levels were detected by Western blot analysis. **(c)** After cells were treated with GSK2334470 and DMSO, the expression levels of VEGFR2 were tested and verified by Western blot analysis.** (d)** The proliferation of HUVECs cultured with TCM in the presence of GSK2334470 was determined by CCK-8 assays. **(e)** Tube formation by HUVECs treated with GSK2334470 was measured, and the results are expressed as the tubule length. Representative statistical results are shown.** (f)** Representative morphological images are shown. Scale bar, 500 µm. ns: no significance, **p <0.01, ***p<0.001.** (g)** Schematic diagram representing the role of TIPE and VEGFR2 in tumor angiogenesis in CRC.

To further elucidate the mechanism by which TIPE regulates VEGFR2 expression and affects angiogenesis, we selected the most studied signaling pathway in the angiogenesis of VEGFR2 according to KEGG and STRING analyses: the PI3K-AKT signaling pathway. We performed Western blot to detect several key kinases in the PI3K-AKT signaling pathway in shTIPE HCT116 cells and shRNA control HCT116 cells. The results showed that knocking down TIPE expression could inhibit RAS expression (Supplementary Figure 1f), further enhancing shTIPE-mediated inhibition of PDK1 phosphorylation (Figure 4a). Subsequently, we treated cells with a PDK1 inhibitor (GSK2334470) and found that there was no difference in TIPE expression before and after treatment. PDK1 phosphorylation in the TIPE control group was inhibited, indicating that PDK1 is a regulatory molecule downstream of TIPE and that TIPE can regulate PDK1 phosphorylation (Figure 4b). In addition, we examined VEGFR2 expression and found that it was lower in shTIPE cells than in control cells and it was further inhibited by a PDK1 inhibitor (Figure 4c).

To determine whether TIPE regulates CRC angiogenesis through PDK1, we used different concentrations of the PDK1 inhibitor in shTIPE HCT116 and shRNA control HCT116 cell cultures and then collected the respective TCM. The collected TCM was used to culture HUVECs which were analyzed by CCK-8 assays to detect their proliferation ability. The results showed that inhibiting PDK1 expression could weaken the proliferation of HUVECs cultured in the supernatant from the shTIPE group (Figure 4d). We then repeated the angiogenesis experiment, and HUVECs were cultured in different conditioned media in the presence of the PDK1 inhibitor. According to statistical analyses of the total branch lengths, there was no statistically significant difference in the total branch lengths between the shTIPE group and the control group after interfering with PDK1 expression (Figure 4e, f), indicating that the cells lost their angiogenic ability. TIPE promoted VEGFR2-mediated angiogenesis by upregulating PDK1 expression and phosphorylation (Figure 4g).

## Discussion

Angiogenesis is the process encompassing the formation of new blood vessels from pre-existing blood vessels and plays an important role in tumor growth, invasion and metastasis 29. Angiogenesis is primarily regulated by VEGF, which binds to VEGFR2 and induces receptor dimerization and phosphorylation 32, 36, 37. Several studies have highlighted the role of VEGF in cancer, particularly in stimulating angiogenesis, and, as a result, many antiangiogenic drugs have been developed for cancer treatment 38. Although the initial response of tumor cells to antiangiogenic drug therapy was a decrease in the tumor size, most of these drugs did not improve overall survival due to the development of drug resistance 39. Angiogenesis is a complex process in the cancer microenvironment. Many genes are reported to be involved in angiogenesis in different tumor types. In addition, complex gene-gene and gene-microenvironment interactions further influence the role of tumor angiogenesis, not just the key molecule VEGF alone. Thus, the complexity requires a search for a new way to regulate VEGF-mediated angiogenesis.

The TNFAIP8 family is a newly discovered family of immune and tumor regulatory factors, among which TNFAIP8/TIPE was the first novel protein discovered in this family and acts as an antiapoptotic and oncogenic molecule 13, 14. In the latest studies of clinical samples, high TIPE expression is correlated with metastasis of endometrial cancer 24. TIPE overexpression is also associated with lymph node metastasis and poor prognosis in intestinal-type gastric adenocarcinoma 40. TIPE is a potential predictor of lymph node metastasis in pN0 esophageal squamous cell carcinoma after Ivor Lewis esophagectomy 41. TIPE promotes the metastasis of non-small-cell lung cancer, mediates cisplatin resistance and can be used as a predictor of poor prognosis 42. These studies indicate that TIPE plays a key role in tumor metastasis and certain types of resistance to chemotherapy. CRC currently has the highest mortality rate due to metastasis, so we attempted to determine both the role of TIPE in CRC metastasis and whether it involves the key step of angiogenesis for metastasis, which affects the poor prognosis of CRC. We speculate that TIPE may act as a major mediator to control the hub of the angiogenesis regulation network (VEGF-VEGFR) and may serve as a better target for tumor treatment.

To date, there have been few reports on TIPE and tumor angiogenesis. This study used clinical samples from 55 patients surgically diagnosed with CRC and found that TIPE levels were higher in the cancer tissues than in the adjacent tissues. Because VEGFR2 is an important mediator of cell migration and angiogenesis, we decided to focus on it and found increased VEGFR2 expression in CRC samples. Later, in vitro and in vivo experiments confirmed that TIPE plays an important role in CRC cell migration and angiogenesis and regulates VEGFR2 expression. We stably transfected HCT116 cells with lentivirus containing shTIPE or shRNA control and collected supernatants from the resulting cells to use as conditioned medium to stimulate HUVECs. After TIPE expression was knocked down in HUVECs, the results of the CCK-8 and angiogenesis assays showed slowed proliferation and failed tubular structure formation, respectively. Then, stable HCT116 cell lines were used for the microfilament IF experiment; compared to the shRNA control HCT116 cells, shTIPE HCT116 cells exhibited a reduced number of pseudopods and weakened migration. At the same time, VEGFR2 expression in shTIPE HCT116 cells was decreased according to the qPCR results. Furthermore, in vivo CAM experiments with induced neovascularization and matrix gel plug models also support our in vitro observation that TIPE promotes angiogenesis in CRC. These data indicate that TIPE can modulate VEGFR2 expression and promote angiogenesis in CRC.

Previous studies have shown that VEGF-A ligands bind to VEGFR2 to exert various pathophysiological effects on cell proliferation, cell migration, and angiogenesis. These changes involve activation of the PI3K-Akt, MAPK and calcium signaling pathways, leading to activation of the VEGF signaling pathway. In our study, we found that knocking down TIPE downregulates VEGFR2 and PDK1 expression. Upon inhibition of PDK1 expression, VEGFR2 expression and PDK1 phosphorylation were reduced. HUVEC proliferation and angiogenesis were also inhibited by the TCM from cells expressing shTIPE. Therefore, TIPE induces VEGFR2-mediated angiogenesis, at least partially, through the PI3K-Akt pathway (Figure 4g).

In summary, this study found for the first time that the expression levels of TIPE and VEGFR2 are upregulated in CRC, which further leads to CRC metastasis by promoting angiogenesis. In addition, TIPE may be involved in the PI3K-Akt pathway to induce VEGFR2-mediated angiogenesis. Overall, our study suggests that targeting TIPE and VEGFR2, both of which participate in CRC angiogenesis, is a very promising and potential method for CRC therapy.

## Materials and Methods

### Cell culture

The CRC cell lines HT29, HCT116, SW480, and SW620 and human embryonic kidney (HEK) 293T cells were obtained from the Cancer Research Center of Xiamen University (Xiamen, Fujian, China). HUVECs were obtained from Procell Life Science & Technology Company. All cells were authenticated by STR profiling according to the cell bank. HUVECs were cultured in F12 Kaighn's medium (F12K, Gibco, Palo Alto, CA), and all other cell lines were cultured in Dulbecco's modified Eagle's medium (DMEM, Gibco) supplemented with 10% fetal bovine serum (FBS, Gibco), 100 units/ml penicillin, and 100 mg/ml streptomycin (Invitrogen, Carlsbad, CA, USA) and maintained under standard conditions (5% CO2 and 95% atmosphere, 37°C).

### Cell transfection

Stably transfected cells were created with a lentiviral system. Lentiviral vectors encoding human shTIPE and an shRNA vector (pSIREN-RetroQ, shcontrol) were donated by Professor Jin Guanghui, School of Medicine, Xiamen University. We used 293T and HCT116 cells in the logarithmic growth phase for the experiments. Retroviruses were prepared in 293T cells by transfecting the target plasmid shTIPE and empty vector Retro-Q using transfection reagent (jet PRIME, Polyplus, Strasbourg, France). Then, the target HCT116 cells were transfected. After 48 hours of transfection, stable cell lines were selected by continuous screening for 6 days with 6 ng/ml puromycin (Sigma-Aldrich, St. Louis, USA), and the screening dose was halved after two weeks. The knockdown and corresponding empty vector cells were cultured for both in vitro and in vivo experiments.

### Preparation of conditioned medium

TCM was prepared by transfecting HCT116 cells under different culture conditions as described in previous studies. The transfected cells were plated in 6-well plates and allowed to adhere to the bottom of the wells. After 24 hours, the medium was removed, and the cells were washed with phosphate-buffered saline (PBS) twice to remove serum components. Then, the medium was replaced with serum-free DMEM (Gibco, Palo Alto, CA), and the cells were incubated for another 24 hours. Later, the supernatants were collected and centrifuged at 1000 rpm for 10 minutes to remove cell debris. To completely separate cells from the TCM, the collected TCM supernatants were centrifuged at 12000 rpm for 10 minutes and filtered through a 0.22 µm filter (Corning Inc., NY, USA). The supernatants were collected and used as TCM for further study.

### Cell viability assays

Cell proliferation was performed using a Cell Counting Kit‐8 (Beyotime, Haimen, China) according to the manufacturer's instructions. Cells were seeded into a 96-well plate (Corning Inc., NY, USA) at an initial density of 3000 cells/well in triplicate. The absorbance at 450 nm was measured at every indicated time point at least three times using a Bio-Rad microplate reader.

### Western blotting and antibodies

Cells were harvested and prepared using RIPA buffer (Sigma-Aldrich, St. Louis, USA) supplemented with 1% protease inhibitor cocktail and 1% phenylmethanesulfonyl fluoride (Gold Biotechnology, USA) at 4°C and were collected by centrifugation. Protein concentrations were determined by the Bradford assay (Bio-Rad, Hercules, CA). Equal amounts of protein (10-40 µg) were separated by SDS-PAGE and transferred to PVDF membranes (Millipore, Billerica, MA, USA). The membranes were washed and incubated at 4°C overnight with the following specific primary antibodies: rabbit polyclonal antibody against human TIPE (1:300; Boster, Wuhan, China), rabbit monoclonal antibody against VEGFR2 (1:1000; Abways, USA), rabbit monoclonal antibody against RAS (1:5000, Abcam, Kendall Square, Suite Cambridge, USA), PDK1, p-PDK1 (1:2000; Millipore, CA, USA), and β-actin (1:1000; ZSGB-Bio, Beijing, China). The next day, the membranes were washed and incubated with horseradish peroxidase (HRP)-conjugated goat anti-mouse IgG and goat anti-rabbit IgG (1:2000; ZSGB-Bio, Beijing, China) antibodies for 1 hour at room temperature. Then, immune-reactive bands were visualized using a BIO-RAD ChemiDoc XRS+ Detection System (Bio-Rad, Hercules, CA) or the traditional darkroom method and quantified by densitometric analysis using a Versadoc imaging system (Bio-Rad, Hercules, CA).

### Quantitative real-time PCR

Surgically resected CRC tissues were obtained from 56 patients at the Department of Gastrointestinal Surgery, Zhongshan Hospital, Xiamen University. All human samples were obtained with informed consent, and all experiments and procedures involving these samples were approved by the Ethics Committee of Zhongshan Hospital, Xiamen University. Total RNA was extracted from tissues or cells using TRIzol reagent (Invitrogen, Carlsbad, CA, USA) according to the manufacturer's instructions. One microgram of RNA was reverse transcribed to cDNA using a Rever-Tra Ace qPCR Kit (Toyobo, Osaka, Japan). Real-time PCR was performed using UltraSYBR Mixture (CWBIO, Beijing, China), and data collection was performed on a Bio-Rad Biosystems 7500 instrument with SYBR Green (Bio-Rad, Hercules, CA). The sequences of the forward and reverse primers were as follows: TIPE-F: 5'-TTCAGGCCTCCCTCTT-TAACAATC-3, TIPE-R: 5'-CGTTCGTGGCAGGGGTTATT-3; VEGFR2-F: 5'-CGGACAGTGGTATGGTTCTTGC-3, VEGFR2-R: 5'-GTGGTGTCTGTGTCATCGGAGTG-3; and β-actin-F: 5'-AGCGAGCATCCCCCAAAGTT-3, β-actin-R: 5'-GGGCACGAAGGCTCATCATT-3. Relative gene expression levels were normalized to those of β-actin, which served as a control.

### Matrigel tube formation experiment

We coated precooled 96-well plates with Matrigel (50 μl/well, BD Biosciences, CA, USA), which was allowed to polymerize at 37 °C for min. Then, HUVECs at a density of 15000 cells per well were cultured in TCM as described in previous studies. After incubation for 6 h at 37 °C, the tube structures were photographed through microscopy, and the tube length was measured with Image-Pro Plus software (Media Cybernetics, L. P, Silver Spring, MD, USA) and expressed as the total length (mm) per field for each well.

### Immunofluorescence for F-actin staining

For IF analysis, shTIPE and control HCT116 cells were grown on coverslips (JingAn Biological, Shanghai, China) in complete medium under basal conditions. All cells were fixed with preheated 4% paraformaldehyde (Sangon, Shanghai, China) for 10 minutes and then washed three times with PBS. Cells were permeabilized with 0.5% Triton X100-PBS at room temperature for 10 minutes and then treated with a sealing solution prepared in 1% BSA in PBS for 5 minutes at room temperature four times to block nonspecific binding. For F-actin staining, we stained coverslips with tetramethylrhodamine (TRITC)-conjugated phalloidin (Sigma-Aldrich, St. Louis, USA) for 1 hour, and nuclei were stained with 4',6-diamidino-2-phenylindole (DAPI) (Beyotime, Haimen, China) for 5 minutes at room temperature. The stained slides were analyzed under a confocal laser microscope (Carl Zeiss, Oberkochen, Germany).

### Chicken embryo angiogenesis assay

Fertilized native eggs were incubated at 37°C under constant humidity. On the third day (E3), the embryo was examined under an egg-laying apparatus. The air chamber was drawn with a pencil, and the inoculation site was drawn on the side of the embryo. Then, 2-3 ml of ovalbumin was gently sucked out of the egg with a needle, and an air sac was formed directly over the CAM to dissociate it from the eggshell membrane. shTIPE and control HCT116 cells (10^5^ cells/100 μl) were mixed with an equal volume of high-concentration Matrigel and then implanted into the CAM. Images of the CAM were captured 7 days after implantation, and two observers counted the branches of the blood vessels in a double-blind manner. The assay for each treatment was performed using 5 chicken embryos.

### In vivo Matrigel angiogenesis assay

BALB/c nude mice (4-5 weeks of age, male) were obtained from the Xiamen University Laboratory Animal Center (Xiamen, Fujian, China) and maintained under pathogen‐free conditions for 1 week. The experiments were performed in accordance with the guidelines of the Animal Care and Use Committee and Ethics Committee of Xiamen University. To explore the angiogenesis-promoting role of TIPE in vivo, shTIPE or control HCT116 cells (3× 10^6^ cells/mouse) were mixed with an equal volume of high-concentration Matrigel and then subcutaneously injected into the flanks of nude mice (n = 5 for each group). At 9 days after cell implantation, the mice were sacrificed, the tumors were removed and weighed, and the lungs and lymph nodes were removed and formalin fixed for HE staining. Histological sections mounted on slides were immunostained with monoclonal antibodies targeting the endothelial cell marker CD31 (1:800; Proteintech Group, Colorado, USA).

### Immunohistochemical analysis

Surgically resected CRC tissues were obtained from 56 patients at the Department of Gastrointestinal Surgery, Zhongshan Hospital, Xiamen University. A total of 56 formalin-fixed, paraffin-embedded CRC specimens were immunostained according to a manual method using a custom-made and validated anti-TIPE antibody (1:50; BOSTER, Wuhan, China) and a VEGFR2 antibody (1:50; Abways, USA) as described previously. We used Image-Pro Plus 6.0 (Media Cybernetics, Inc., Rockville, MD, USA) to analyze the immunohistochemical staining density and average optical (AO) density. The CD31 staining pattern was semiquantitatively assessed based on the staining intensity and distribution, and the results were correlated with morphologic and prognostic variables.

### Statistical analysis

All statistical analyses were performed using GraphPad Prism 5 software (San Diego, CA) and SPSS 13.0 software (Chicago, IL, USA). Quantitative data are expressed as the mean ± S.D. Significant differences for quantitative data were compared by two-tailed Student's t-test. The significance of the correlation between the expression of the indicated proteins and histopathological factors was determined using the Pearson χ2 test. In all samples, p < 0.05 was considered to be significant. Statistical significance is indicated as *p<0.05, **p<0.01, ***p<0.001, and **** p < 0.0001.

## Supplementary Material

Supplementary figures and tables.

## Figures and Tables

**Table 1:** Distribution of tumor necrosis factor-α induced protein 8 (TIPE) expression in colorectal cancer patients according to clinicopathological characteristics

Characteristics	TIPE		χ^2^	P value
	Positive	Negative		
**Gender**				
Male	20	18	0.00045	0.98304
Female	9	8		
**Age**				
≥65Y	11	9	0.06513	0.79857
<65Y	18	17		
**The depth of invasion**				
Over muscularis propri	27	19	4.0174	**0.04503**
Less than muscularis propri	2	7		
**Lymph nodes involvement**				
Yes	16	4	9.37855	**0.0022**
No	13	22		
**Distant metastasis**				
Yes	5	1	2.53097	0.11163
No	24	25		
**Differentiation**				
Moderate	23	21	0.01824	0.89258
Poor	6	5		
