# Supplementary material for: TIPE regulates VEGFR2 expression and promotes angiogenesis in colorectal cancer
Source: Int J Biol Sci. 2020 Jan 1;16(2):272–83. doi: 10.7150/ijbs.37906 (PMC6949158; doi:10.7150/ijbs.37906)
Supplement: Supplementary file 1 — Supplementary figures and tables. [file ijbsv16p0272s1.pdf]

Supplementary Material

Supplementary Figure 1. Histograms demonstrate mRNA upregulation in CRC samples relative to that in normal samples (data downloaded from Oncomine). **(a)** The immunohistochemical density of TIPE expression as assessed by statistical analysis. **(b)** Relative TIPE mRNA expression in Sabates-Bellver datasets, which include 32 matched pairs of colorectal carcinoma and normal colon samples. **(c)** Detection of relative VEGFR2 mRNA expression in the same database. **(d)** The linear analysis relationship was positively correlated. **(e)** The immunohistochemical density of VEGFR2 expression as assessed by statistical analysis. **(f)** The expression levels of RAS in shTIPE and shRNA control HCT116 cells were determined by Western blot analysis. ns: no significance, $^{**}p < 0.01$, $^{****}p < 0.0001$.

Supplementary Table 1. Difference between TIPE and VEGFR2 overexpression in colorectal cancer.

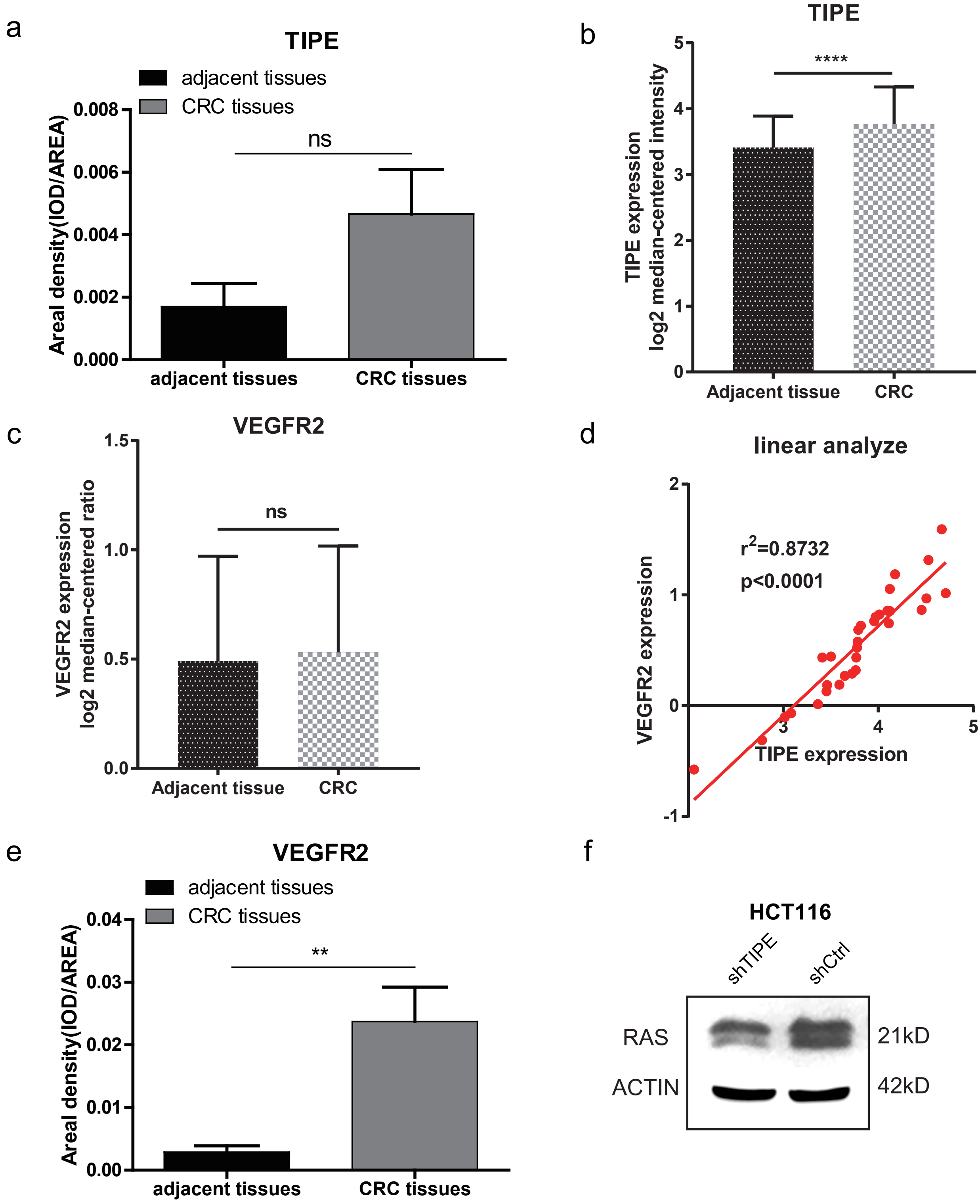
a
TIPE
adjacent tissues
CRC tissues
Areal density(IOD/AREA)
0.008
0.006
0.004
0.002
0.000
ns
adjacent tissues
CRC tissues
b
TIPE
TIPE expression
log2 median-centered intensity
5
4
3
2
1
0
****
Adjacent tissue
CRC
c
VEGFR2
VEGFR2 expression
log2 median-centered ratio
1.5
1.0
0.5
0.0
ns
Adjacent tissue
CRC
d
linear analyze
r^2=0.8732
p<0.0001
VEGFR2 expression
TIPE expression
2
1
0
-1
3
4
5
e
VEGFR2
adjacent tissues
CRC tissues
Areal density(IOD/AREA)
0.04
0.03
0.02
0.01
0.00
**
adjacent tissues
CRC tissues
f
HCT116
shTIPE
shCtrl
RAS
ACTIN
21kD
42kD

Supplementary Table 1. Difference between TIPE and VEGFR2 overexpression in colorectal cancer.

		TIPE		
		+	−	sum
VEGFR2	+	24	7	31
	−	5	19	24
	sum	29	26	55

χ^2= 17.3773，P<0.001
